# Differentiated Service Delivery Model in Improving HIV Treatment Outcomes Among Female Sex Workers in Gauteng Province of South Africa: A Protocol Paper

**DOI:** 10.3390/mps7060089

**Published:** 2024-11-01

**Authors:** Lifutso Motsieloa, Edith Phalane, Refilwe N. Phaswana-Mafuya

**Affiliations:** 1 South African Medical Research Council/University of Johannesburg (SAMRC/UJ) Pan African Centre for Epidemics Research Extramural Unit (PACER EMU), Johannesburg 2092, South Africa; lifutso@gmail.com (L.M.); edithp@uj.ac.za (E.P.); 2 Faculty of Health Sciences, University of Johannesburg, Johannesburg 2092, South Africa

**Keywords:** HIV treatment, differentiated service delivery, female sex workers, people living with HIV, South Africa

## Abstract

South Africa developed the differentiated service delivery (DSD) model to improve access to healthcare for people living with HIV (PLHIV), especially key populations (KPs) including female sex workers (FSWs) who often face barriers in accessing HIV services. The DSD model, aims to reduce the burden on healthcare users, healthcare workers, and the healthcare system, can significantly benefit this group. However, the success of the DSD model in achieving the desired HIV treatment outcomes for FSWs has been barely evaluated. This paper describes the protocol for evaluation of the DSD model in improving HIV treatment outcomes among FSWs in Gauteng Province of South Africa. Both qualitative and quantitative methods will be utilized to address three study objectives: stakeholder analysis, mapping, and in-depth interviews (objective 1); programme evaluation of the DSD model in selected sites (objective 2); and development of a framework for optimizing the DSD model in improving HIV treatment outcomes (objective 3). Quantitative statistical analysis will be performed using STATA version 17 (College Station, TX, USA). Qualitative analysis will be performed using ATLAS.ti. This study will provide new insights into the utilization of the DSD model among FSWs in South Africa. It will also inform new strategies for the DSD model’s implementation in the country. This study will contribute towards the development of a framework for strengthening the DSD model in improving HIV treatment outcomes among FSWs in Gauteng Province.

## 1. Introduction

South Africa has a generalized human immunodeficiency virus (HIV) epidemic, with an estimated 8 million people living with HIV (PLHIV) in 2022, and this equates to a total HIV prevalence of 13.2% with more women affected than men [1,2]. The number of new HIV infections was estimated at 164,228 in 2022, reflecting a decline across all age groups and in both females and males since 2016 [1]. The prevalence of HIV among female sex workers (FSWs) in South Africa is projected to be in the range of 39–89% in different locations of the country [3]. Most recently, the Thembisa model estimated the prevalence of HIV among FSWs at 58,8% [1]. Additionally, Kassanjee et al. [3] reported an HIV prevalence of 62% among FSWs. The heightened HIV risk among FSWs is exacerbated by punitive laws that increase vulnerability, pushing them into unsafe environments and intensifying economic hardships, while substance and alcohol abuse, coupled with multiple sexual partners, further compound these risks [4,5]. South Africa is dedicated to achieving the Joint United Nations Programme on HIV/AIDS (UNAIDS) 95-95-95 targets by enhancing HIV prevention, treatment, and care. The country aims to eliminate HIV as a public health issue by 2030 by addressing barriers and promoting fair access to services [4]. South Africa is currently falling short of the second 95 target, which seeks to have PLHIV receive treatment. So far, only 80% of the general population has met this target, with even lower rates among key populations (KPs), according to data from the District Health Information System (DHIS) that is collected by the National Department of Health (NDoH) [1,6].

South Africa has the largest antiretroviral therapy (ART) programme globally, providing treatment to around 5.6 million people living with HIV (PLHIV). Among these, approximately 3.4 million individuals are receiving first-line ART, 145,000 are on second-line ART (SLART), and over 700 are utilizing third-line ART (TLART) [7]. In order to improve HIV treatment and care in PLHIV, the differentiated service delivery (DSD) model, also known as the “patient-centered” strategy, was implemented and has become an integral component of the HIV cascade programmes [8,9]. It consists of four building blocks, namely *When is care provided? Where is care provided? Who provides the care? And what care or services are provided?* [10]. The DSD model is an approach that offers a more adaptable and patient-focused approach to HIV care by customizing services to the diverse needs of different populations [10]. It facilitates the delegation of tasks to community healthcare workers and provides care in more convenient locations, such as community-based settings, rather than limiting it to traditional clinics. Notable features of the DSD model include longer medication refill intervals, reduced frequency of follow-up appointments, support for medication adherence, and the integration of various health services. Additionally, it emphasizes the vital importance of involving the community in providing care [8,11].

Traditionally, South Africa’s HIV care system used a uniform approach where all patients followed the same treatment plans and visited clinics frequently, regardless of their individual needs. This system caused problems, especially for KPs like FSWs and people in remote areas, leading to high drop-out rates, poor treatment adherence, and suboptimal viral suppression [11]. In improving the DSD model, South Africa shifted care to local clinics, giving more responsibility to nurses and community health workers, and offering multi-month prescriptions for stable patients. These strategies alleviated clinic overcrowding and improved patient retention by making healthcare more accessible and personalized [11,12,13]. However, challenges remain, including linking KPs to care, improving rural infrastructure, and integrating HIV treatment with other conditions like tuberculosis and chronic diseases [4].

In settings with limited resources and high demands of PLHIV, the DSD model is being implemented to reduce visits to health facilities for HIV services. For instance, ART refills are frequently separated from clinical visits in the DSD models. Due to COVID-19 restrictions and lockdowns, national HIV policies swiftly adapted, expanding eligibility for decentralized HIV treatment, prolonging ART refills, introducing virtual care models, and enhancing community-based treatment approaches [14]. These approaches included extending ART refills, using telehealth for follow-ups and support, and employing community-based strategies.

The above adaptations have been implemented within the general population due to the absence of disaggregated data on key populations (KPs) in the DHIS. However, a study by Wang et al. [15] showed that the Centralized Chronic Medication Dispensing and Distribution (CCMD) system effectively supported differentiated care for clinically stable PLHIV. The PLHIV participating in the programme maintained high rates of viral suppression and retention, showing that community-based ART delivery did not negatively affect their treatment outcomes [15].

In a separate study, the “Teen Club” programme, which focused on adolescents accessing ART through a youth-centered differentiated care model, reported higher attrition compared to standard care [16]. While most DSD models have been designed for the general population, they often lack specific data on HIV outcomes for KPs, limiting the reporting on how effective these models are in improving treatment for groups FSWs. Additionally, coverage of these models for FSWs remains lower compared to the general population.

This paper aims to describe the protocol for evaluating the DSD model in improving HIV treatment outcomes among FSWs for the decentralization of HIV care in South Africa. By outlining the objectives and methodology in a rigorous, standardized approach, the protocol serves to inform stakeholders, foster collaboration, and establish a baseline for future assessments. This is essential for advancing evidence-based strategies that enhance HIV treatment access and outcomes for FSWs.

### Research Questions

The proposed study will answer the following questions:

○ What are the experiences, challenges, opportunities, and successes in the implementation of the DSD model by stakeholders in Gauteng Province in South Africa?
○ Does the DSD model enhance the achievement of HIV treatment outcomes among FSWs in South Africa?
○ How is the DSD model being utilized among FSWs in Gauteng Province? How are the HIV clinical outcomes of FSWs monitored?

## 2. Methods

The proposed study will follow a mixed-methods approach involving stakeholder analysis, mapping, and in-depth interviews with stakeholders on the DSD model (objective 1); programme evaluation of the DSD model in selected sites in Gauteng Province (objective 2) as well as developing a framework for the optimization of the DSD model in improving HIV treatment services among FSWs (objective 3).

### 2.1. Methods for Objective 1

To identify and solicit the views of stakeholders regarding their interests, successes, challenges, improvements, and experiences in the implementation of the DSD model in South Africa.

This objective will comprise of stakeholder analysis, mapping, and in-depth-interviews with stakeholders.

#### 2.1.1. Methods for Stakeholder Analysis and Mapping

The stakeholder analysis and mapping is a method used to identify which stakeholders to engage, their interests and relevance, as well as inter-relationships [16]. In this regard, a stakeholder analysis and mapping will be conducted to identify and prioritize stakeholders, and assess their interests, successes, challenges, improvements, and experiences in the implementation of the DSD model in South Africa. This method will be carried out in four stages: defining the stakeholders in the context of the DSD model (Stage 1); understanding the stakeholder’s interest/relevance to the DSD model (Stage 2); assessing stakeholder’s influence on the DSD model (Stage 3); and developing strategies for engagement (Stage 4) [17].

Stage 1—Define the stakeholders involved in the implementation of the DSD model: A snowballing method will be used to identify key stakeholders by asking individuals from both governmental and non-governmental organizations (NGOs) to recommend people they consider influential in implementing the DSD model among FSWs in South Africa. Stakeholders will be selected from various sectors involved in the HIV care continuum, including prevention, treatment, care, and support services, as well as those directly linked to the DSD model. Organizations of interest will include government health and social departments, NGOs, community-based organizations (CBOs), implementing partners, civil society organizations (CSOs), United Nations agencies, and multilateral funding bodies.

Stage 2—Understanding stakeholders’ interests with regards to the DSD model: a list of potential stakeholders will be created based on the information gathered in Stage 1.

Stage 3—Assess stakeholders’ influence on the implementation of the DSD model: This step will involve evaluating the stakeholders’ influence on the project and its outcomes. A four-quadrant matrix will be used to map out each stakeholder’s level of interest and influence in the DSD model’s implementation.

Stage 4—Developing strategies for engagement: communication strategies will be developed, which may include creating communication plans, organizing meetings, or establishing incentives to ensure stakeholder engagement [17].

#### 2.1.2. Methods for In-Depth Interviews with Stakeholders

##### Study Design

An exploratory study design will be employed to obtain in-depth information about the subject matter.

##### Study Population

The study population will be drawn from the list of stakeholders identified through the stakeholder analysis and mapping in objective 1. Stakeholders will comprise policymakers, executives, coordinators, supervisors or managers, advisory, informing and advocacy roles, FSWs, and PLHIV involved in different areas (i.e., HIV prevention, treatment, care, and other supportive interventions) of the DSD model.

##### Sample

A purposive sample of 15-20 stakeholders aged 18 years and above will be recruited to gather rich-textured information on the DSD model [18]. The sample size may change depending on when saturation is reached, i.e., when no new information is received from stakeholders. Stakeholders who are willing to voluntarily grant written informed consent and are working in the selected study area will be included to take part in the in-depth interviews. Additionally, consent will be sought for audio recording from the stakeholders.

##### Recruitment of Participants

The implementing partners will link the researcher to the gatekeepers or managers, who will in turn assist with inviting potential stakeholders to participate in this study. Stakeholders will be invited to attend information sessions at implementation sites, where they will receive a study information letter outlining this study’s objectives, participation expectations, and the researcher’s contact details. Alternatively, the information letter may be emailed to potential stakeholders. Since the implementing partners and managers hold access to stakeholders’ personal and contact information, they will be responsible for sharing the study details with them. Stakeholders interested in participating in the in-depth interviews will reach out to the researcher to arrange a convenient time, date, and location for the interview.

##### Data Collection

Face-to-face, in-depth interviews will be carried out with stakeholders using a set of guiding questions. These questions will concentrate on the current DSD model, initiatives, structures, and relevant policies regarding priority areas, needs, gaps, and opportunities. The guiding questions will be tailored to the specific stakeholders and FSWs. Field notes will be recorded during the interviews to supplement the audio data in case of equipment malfunction. The interviews will conclude once data saturation is reached or if the stakeholders choose not to continue.

##### Data Analysis

The data will be captured on ATLAS.ti version 22 (Scientific Software Development GmbH, Berlin) or related software. Inductive coding will be used and generated from ATLAS.ti or related software [19]. Before data analysis is performed, the interviews will be transcribed. Thereafter, the dataset will be read to come up with codes. ATLAS.ti’s inter-coder agreement will be used to deal with discrepancies [19]. Themes and sub-themes will be created using the data (thematic content analysis).

### 2.2. Methods for Objective 2

To conduct programme evaluation of the DSD model in improving HIV treatment outcomes among female sex workers in selected facilities in Gauteng Province.

#### 2.2.1. Study Design

A participatory evaluation approach using both quantitative and qualitative methods will be utilized.

#### 2.2.2. Study Population

The target population will be the Department of Health (DoH) implementing partners that have executed the DSD model for HIV treatment services among FSWs in Gauteng Province. Implementing partners affiliated with the DoH, who have been providing HIV treatment services for at least a year and have a gatekeeper permission in place, will be included. The inclusion criteria for the DSD model implementors, coordinators/managers, internal programme staff, FSWs, and external stakeholders will be those aged 18 years and above, providing HIV treatment services for at least a year or who have utilized HIV treatment services in the last year, knowledgeable on the DSD model for HIV treatment services, as well as willing to take part in the study.

#### 2.2.3. Sample

A purposive sampling procedure will be utilized to select four to six primary implementing sites. Within each implementing sites, 10 DSD model implementors, coordinators/managers, programme staff, FSWs, and external stakeholders will be interviewed. In total, 40 individuals will be interviewed across the four primary implementing partners sites.

#### 2.2.4. Data Collection

Inventory of services will be completed using a questionnaire informed by the Consolidated Framework for Implementation Research (CFIR) 2.0 [20]. The questionnaire will include open-ended questions, to gain insights about the DSD model resources and personnel, and close-ended questions. The qualitative component will be reported following the Consolidated Criteria for Reporting Qualitative Research (COREQ) to ensure thoroughness and precision [21]. For the quantitative component, the reporting will be guided by the Strengthening the Reporting of Observational Studies in Epidemiology (STROBE) checklist [22]. Data will be collected on the DSD model activities and components, including barriers and facilitators, services, implementation, [20] and HIV treatment outcomes (see Table 1).

#### 2.2.5. Data Source

De-identified data will be requested from the provincial DoH implementing partners that have implemented the DSD model for HIV treatment uptake among FSWs in Gauteng Province. The data will include programme data, reports, and other pertinent documents related to the implementation of the DSD model. The focus will be on data collected from January 2019 and onwards. The changes in service, implementation, and HIV treatment outcomes will be compared before and after the implementation of the DSD model.

This evaluation will provide information on whether the DSD model is achieving the desired outcomes and identify opportunities for quality improvement, optimal utilization of resources, and support for staff and managers to improve the programme and make modifications as needed. This will be used to further strengthen programme effectiveness and future development.

#### 2.2.6. Data Analysis

Thematic content analysis will be performed for the data obtained using the open-ended questions. Themes and sub-themes will be formulated in line with the CFIR 2.0 constructs [20]. Analysis will be completed using ATLAS.ti software.

Descriptive analysis will be performed and reported as proportions (n) and frequencies (%) using the latest version of STATA software version 17 (College Station, TX, USA). An interrupted Time series analysis will be completed to assess the changes in service, implementation, and HIV treatment outcomes (Table 1) before (April 2019) and after (January 2020 and onwards) the implementation of the DSD model. The DSD model will serve as the interruption, and comparison will be conducted for two periods (before and after the DSD model).

### 2.3. Methods for Objective 3

To develop a framework for optimization of the DSD model, synthesizing results from objectives 1 and 2.

The development of a framework for optimizing the DSD model in improving HIV treatment outcomes will include the triangulation and synthesis of information from objectives 1–3. This will be guided by the WHO building blocks and analysis of the four building blocks in terms of when, where, who, and what will be [10]. The WHO building block will be used to synthesis the results of objectives 1 to 2 to construct a framework for optimizing the DSD model, building on the evidence provided by the implementation of the WHO building blocks in defining “*When refers to the frequency of service delivery, Where refers to the place of service delivery, Who refers to the person providing the service and What is regarding the services offered*” [10].

## 3. Ethics Considerations

This study is part of a doctoral study by the first author which has attained University of Johannesburg (UJ) Higher Degrees Committee and Research Ethics Committee (REC) approval (REC-2519-2024). This study is also part of the South African Medical Research Council/University of Johannesburg—Pan African Centre for Epidemics Research (PACER) Extramural Unit’s funded umbrella research projects, namely “Harnessing big heterogeneous data to evaluate the potential impact on HIV responses among the key population in generalized epidemic settings in SSA (REC-1504-2022)”; and “Epidemiologic analyses of the impacts of COVID-19 to inform tailoring and adaptation of implementation strategies for HIV service delivery among key populations in Sub-Saharan Africa (REC-1781-2022)”, which also obtained ethics approval.

This study will adhere to the highest ethical standards and norms to guide the involvement of participants in this study. Prior to participating in this study, individuals will receive an information letter outlining this study’s objectives and procedures. All participants will be required to provide written informed consent and sign an anonymous privacy declaration form. This process will include the collection of personal information in accordance with the Protection of Personal Information Act (POPIA). With the participants’ consent, interviews will be audio-recorded. Participants will be informed that their involvement in this study is entirely voluntary and that they have the right to withdraw from this study at any point before the interview begins, without any negative consequences.

Several measures will be implemented to ensure the secure use of de-identified secondary data, minimizing the risk of data loss and unauthorized access. De-identified data transfer will occur through secure channels that provide restricted, time-limited access, protected by passwords. The data will be shared with the researcher after securing a data sharing agreement to access and analyze de-identified programme data. This agreement will comply with the POPIA and the University of Johannesburg’s data management policy.

All interview recordings will be stored securely, in accordance with the previously established data management plan. During document reviews, identifying information such as names, personal identifiers, passport numbers, contact details, or addresses will be removed to protect sensitive data. The POPIA defines personal information, including race, employment history, and geographic location. For the purposes of this study, collecting such information will deepen the understanding of participants’ experiences and challenges in implementing the DSD model.

## 4. Discussion

There are no evaluations of the DSD model for HIV treatment among FSWs in South Africa, especially during the COVID-19 pandemic. Female sex workers continue to experience barriers in accessing HIV services in the public health domain due to structural (stigma and discrimination) and legal factors [4,5]. South Africa under-performed in terms of having 95% of the PLHIV initiated on ART and this was particularly lower for KPs, including FSWs [1,6]. Additionally, at the height of COVID-19, the DSD model was expanded to improve the delivery and utilization of HIV treatment services among HIV patients outside of health facilities, expanding who was eligible for DSD for HIV treatment. Even though there was an expansion of the DSD model during COVID-19, little is known about how the DSD model improved HIV treatment outcomes of FSWs. Furthermore, little is known about how the DSD model is being implemented for FSWs, what successes and challenges are being experienced, as well as the improvements needed. A female sex workers programme implementing the DSD model has not been evaluated using the scientific approach. This approach will enable the researchers to harness experiences of the FSWs, providers, and implementors with the DSD model of care and HIV treatment cascade, which have not been studied. This study will attempt to close gaps that might have contributed to the implementation of the DSD model among FSWs in South Africa. A context-specific framework that can guide HIV services for FSWs will be critical ahead of the 2030 agenda to end HIV as an epidemic.

This study is aligned to the UNAIDS global strategy (2021–2026) for ending inequalities and AIDS as well as the National Strategic Plan (NSP) 2023–2028, Strategic Goal 1 (“Break down barriers to achieving outcomes for HIV, TB and STIs”) [4]. The NSP’s 2023–2028 Strategic Goal 1 looks at removing barriers to service availability, access, and updates in communities through human rights-based, and people and community-centered approaches [4]. Hence, this study aims to evaluate the DSD model in improving HIV treatment outcomes among FSWs for the decentralization of HIV care in South Africa.

## 5. Conclusions

This study will develop a framework for strengthening the DSD model in improving HIV treatment outcomes among FSWs in Gauteng Province. The results of this study will be shared with the implementing partner and other key stakeholders including the DoH. This paper is part of a PhD thesis conducted by article format and the findings of this study may be published in peer-reviewed journals and Department of Higher Education and Training accredited journals, as well as disseminated at scientific conferences/meetings.

This study will also provide new insights into the implementation, access, and utilization of the DSD model in South Africa, especially for FSWs. This study is further likely to inform new strategies for the DSD model’s implementation in the country.

## Figures and Tables

**Table 1:** Service, implementation, and HIV treatment outcome measures.

Outcome Category	Indicator
Service outcomes	“*Accessibility; Coverage; Efficiency; Effectiveness; Efficacy; Quality services; Timeliness; and People-centeredness*” [20]
Implementation outcomes	“*Service acceptability (comfort/relative advantages); Appropriateness (utilization rates on innovation, proportion offered, and accepted); The credibility of implementing programs and policies; Uptake and fidelity; Feasibility (utility and practicality); Durability and reach; Service utilization and availability (rates and timing of utilization of services at different time periods); Scalability sustainability (maintenance and routinization)*” [20]
HIV treatment outcomes	Number/percentage of FSWs: reached; tested for HIV linked to HIV care; initiated on HIV treatment; and adhering to HIV treatment and viral load suppression

## Data Availability

The data generated from objectives 1 and 2 can be shared once available and on request.

## References

[1] Johnson L.F., Meyer-Rath G., Dorrington R.E., Puren A., Seathlodi T., Zuma K., Feizzadeh A. (2022). The Effect of HIV Programs in South Africa on National HIV Incidence Trends, 2000–2019. JAIDS J. Acquir. Immune Defic. Syndr..

[2] Fox M.P., Pascoe S., Huber A.N., Murphy J., Phokojoe M., Gorgens M., Rosen S., Wilson D., Pillay Y., Fraser-Hurt N. (2019). Adherence clubs and decentralized medication delivery to support patient retention and sustained viral suppression in care: Results from a cluster-randomized evaluation of differentiated ART delivery models in South Africa. PLoS Med..

[3] Kassanjee R., Welte A., Otwombe K., Jaffer M., Milovanovic M., Hlongwane K., Puren A.J., Hill N., Mbowane V., Dunkle K. (2022). HIV incidence estimation among female sex workers in South Africa: A multiple methods analysis of cross-sectional survey data. Lancet HIV.

[4] South African National AIDS Concil (2023). National Strategic Plan on HIV, TB and STIs, 2023–2028.

[5] Lyons C.E., Schwartz S.R., Murray S.M., Shannon K., Diouf D., Mothopeng T., Kouanda S., Simplice A., Kouame A., Mnisi Z. (2020). The role of sex work laws and stigmas in increasing HIV risks among sex workers. Nat. Commun..

[6] National Department of Health (Pretoria, South Africa) 95 95 95 Cascades. DHIS April 2024. Pretoria.

[7] Naidoo K., Ramruthan J., Reddy M., Lancaster R. (2022). A third-line antiretroviral therapy register to track patient clinical and virological outcomes. South Afr. Med. J. Suid-Afr. Tydskr. Vir Geneeskd..

[8] Ehrenkranz P., Grimsrud A., Holmes C., Preko P., Rabkin M. (2021). Expanding the vision for differentiated service delivery: A call for more inclusive and truly patient-centered care for people living with HIV. J. Acquir. Immune Defic. Syndr..

[9] Mukumbang F.C., Mukumbang F.C. (2021). Leaving No Man Behind: How Differentiated Service Delivery Models Increase Men’s Engagement in HIV Care. Int. J. Health Policy Manag..

[10] World Health Organization (2021). Updated Recommendations on Service Delivery for the Treatment and Care of People Living with HIV.

[11] Pascoe S.J., Fox M.P., Huber A.N., Murphy J., Phokojoe M., Gorgens M., Rosen S., Wilson D., Pillay Y., Fraser-hurt N. (2019). Differentiated HIV care in South Africa: The effect of fast-track treatment initiation counselling on ART initiation and viral suppression as partial results of an impact evaluation on the impact of a package of services to improve HIV treatment adherence. J. Int. AIDS Soc..

[12] Bogart L.M., Shazi Z., MacCarthy S., Mendoza-Graf A., Wara N.J., Zionts D., Dube N., Govere S., Bassett I.V. (2022). Implementation of South Africa’s Central Chronic Medicine Dispensing and Distribution Program for HIV Treatment: A Qualitative Evaluation. AIDS Behav..

[13] Long L., Kuchukhidze S., Pascoe S., Nichols B.E., Fox M.P., Cele R., Govathson C., Huber A.N., Flynn D., Rosen S. (2020). Retention in care and viral suppression in differentiated service delivery models for HIV treatment delivery in sub-Saharan Africa: A rapid systematic review. J. Int. AIDS Soc..

[14] Grimsrud A., Ehrenkranz P., Sikazwe I. (2021). Silver linings: How COVID-19 expedited differentiated service delivery for HIV. J. Int. AIDS Soc..

[15] Wang M.M., Violette L.R., Dorward J.M., Ngobese H., Sookrajh Y.M., Bulo E.B., Quame-Amaglo J.M., Thomas K.K., Garrett N.M., Drain P.K. (2023). Delivery of Community-based Antiretroviral Therapy to Maintain Viral Suppression and Retention in Care in South Africa. J. Acquir. Immune Defic. Syndr..

[16] Ginige K., Amaratunga D., Haigh R. (2018). Mapping stakeholders associated with societal challenges: A Methodological Framework. Sci. Direct.

[17] Franco-Trigo L., Fernandez-Llimos F., Martínez-Martínez F., Benrimoj S., Sabater-Hernández D. (2020). Stakeholder analysis in health innovation planning processes: A systematic scoping review. Health Policy.

[18] Vasileiou K., Barnett J., Thorpe S., Young T., Vasileiou K., Barnett J., Thorpe S., Young T. (2018). Characterising and justifying sample size sufficiency in interview-based studies: Systematic analysis of qualitative health research over a 15-year period. BMC Med. Res. Methodol..

[19] Vears D.F., Gillam L. (2022). Inductive content analysis: A guide for beginning qualitative researchers. Focus Health Prof. Educ. A Multi-Prof. J..

[20] Damschroder L.J., Reardon C.M., Opra Widerquist M.A., Lowery J., Damschroder L.J., Reardon C.M., Opra Widerquist M.A., Lowery J. (2022). Conceptualizing outcomes for use with the Consolidated Framework for Implementation Research (CFIR): The CFIR Outcomes Addendum. Implement. Sci..

[21] Tong A., Sainsbury P., Craig J. (2007). Consolidated criteria for reporting qualitative research (COREQ): A 32-item checklist for interviews and focus groups. Int. J. Qual. Health Care.

[22] Von Elm E A.D., Egger M., Pocock S.J., Gøtzsche P.C., Vandenbroucke J.P. (2007). The Strengthening the Reporting of Observational Studies in Epidemiology (STROBE) statement: Guidelines for reporting observational studies. PLoS Med..

